# Thermodynamic Analysis of Chemically Reacting Mixtures—Comparison of First and Second Order Models

**DOI:** 10.3389/fchem.2018.00035

**Published:** 2018-03-01

**Authors:** Miloslav Pekař

**Affiliations:** Institute of Physical and Applied Chemistry and Materials Research Centre, Faculty of Chemistry, Brno University of Technology, Brno, Czechia

**Keywords:** affinity, entropic inequality, independent reactions, kinetics, rate constants, rate equations, thermodynamics

## Abstract

Recently, a method based on non-equilibrium continuum thermodynamics which derives thermodynamically consistent reaction rate models together with thermodynamic constraints on their parameters was analyzed using a triangular reaction scheme. The scheme was kinetically of the first order. Here, the analysis is further developed for several first and second order schemes to gain a deeper insight into the thermodynamic consistency of rate equations and relationships between chemical thermodynamic and kinetics. It is shown that the thermodynamic constraints on the so-called proper rate coefficient are usually simple sign restrictions consistent with the supposed reaction directions. Constraints on the so-called coupling rate coefficients are more complex and weaker. This means more freedom in kinetic coupling between reaction steps in a scheme, i.e., in the kinetic effects of other reactions on the rate of some reaction in a reacting system. When compared with traditional mass-action rate equations, the method allows a reduction in the number of traditional rate constants to be evaluated from data, i.e., a reduction in the dimensionality of the parameter estimation problem. This is due to identifying relationships between mass-action rate constants (relationships which also include thermodynamic equilibrium constants) which have so far been unknown.

## Introduction

Investigating impacts of thermodynamics on kinetics of chemical reactions is an area of unflagging interest and continuous research. Due to the inherent non-equilibrium nature of ongoing chemical reactions, especially the non-equilibrium thermodynamics brings significant progress to the aim to put thermodynamics and kinetics in a common framework (for example, Pagonabarraga et al., 1997; Bedeaux et al., 2010; Kannan and Rajagopal, 2011; Klika and Grmela, 2013; Rubí et al., 2013; Arato and Morro, 2014; Bothe and Dreyer, 2015; Ge and Qian, 2016; N'Guyen et al., 2016).

Recently, a paper was published describing the detailed (non-equilibrium) thermodynamic analysis of chemically reacting mixtures with consequences for kinetic models (Pekař, 2016). A mixture of three isomers was used as an example to illustrate this approach, i.e., a first order kinetic model was analyzed. An anonymous reviewer raised the question of what the effect would be of including a second order reaction (a bimolecular reaction, in this case, but for the purpose of this work the distinction between order and molecularity is not essential; only the term *order* will be used henceforth). Because the answer to this interesting and important question is neither short nor simple, providing it was postponed until this work.

The abovementioned approach (Pekař, 2016) attempts to find some thermodynamic restrictions on chemical kinetics (Boyd, 1977). The majority of these approaches are of an *a posteriori* type and use principles already established in equilibrium thermodynamics (Qian, 2007; Ederer and Gilles, 2008; Al-Khateeb et al., 2009; Vlad and Ross, 2009; Fleming et al., 2010; Hernández-Lemus et al., 2014; Jones et al., 2015). *A posteriori* in this context means that a reaction scheme is designed first and then thermodynamics is applied to corresponding kinetic (rate) equations.

The method of this (and the preceding) paper is of an *a priori* type—it begins only with a list of components of a reacting mixture. Then, the results of non-equilibrium thermodynamics (Pekař and Samohýl, 2014) are combined with the mathematics of stoichiometry (Bowen, 1968) to find independent reactions and, subsequently, their final rate equations in the form of a thermodynamic polynomial. This polynomial is very close to traditional mass-action rate equations. The rate equations are then analyzed for their consistency with a condition emerging from the entropic inequality (the second law) given in more detail below (see Equation 2). The method not only directly derives thermodynamically consistent and sufficient rate equations but also gives constraints on their coefficients (representing, in fact, rate constants) required to ensure consistency with the second law. At this stage of development the method is worked out for a reacting mixture of linear fluids (Pekař and Samohýl, 2014), which, however, comprises many systems of interest to chemists.

The main steps of the method are briefly as follows. First, the number of independent reactions is determined on the basis of the list of components of a reacting mixture and their atomic compositions (Bowen, 1968; Pekař and Samohýl, 2014; Pekař, 2016). Non-equilibrium continuum thermodynamics proves (Samohýl, 1982; Pekař and Samohýl, 2014) that reaction rates (of independent reactions) are functions of concentrations and temperature: **J** = **J**(*T, c*_1_, *c*_2_,…, *c*_*n*_) = **J**(*T*, **c**). This function is approximated by a polynomial in concentrations:

(1) $$J=\sum_{\beta \;=\;1}^{Z}k_{{\vec{\nu }}_{\beta }}\prod_{\alpha \;=\;1}^{n}c_{\alpha }^{\nu_{\beta \alpha }},\;\sum_{\alpha \;=\;1}^{n}\nu_{\beta \alpha }\leq M$$

Here, **J** is the vector whose components are the rates of *R* independent reactions, **J** = (*J*_1_, *J*_2_,…, *J*_*R*_), *c*_*i*_ is the molar concentration of component *i*, and *n* is the total number of components. The vector ${\text{k}}_{{\vec{\nu }}_{\beta }}$ contains polynomial coefficients dependent on temperature only, the vectors ${\vec{\nu }}_{\beta }$ = (ν_β1_,ν_β2_,…, ν_β*n*_) contain polynomial powers and are also used as subscripts to index various vectors of polynomial coefficients (${\text{k}}_{{\vec{\nu }}_{\beta }}$), and *M* is the polynomial degree. For the total number of terms *Z*, see Bowen (1968) and Pekař and Samohýl (2014).

The equilibrium condition is applied and the polynomial is modified to a simplified final form, called the thermodynamic polynomial, which contains also thermodynamic equilibrium constants; no reversed rate constants are used. Although the second law was already applied in the application of results of non-equilibrium thermodynamics mentioned above, there is still a condition resulting from this law which is not used and is ignored in other works. This condition refers to reaction rates expressed as functions of (chemical) affinities (*A*^*p*^) and reads (Pekař and Samohýl, 2014):

(2) $$\sum_{p\;=\;1}^{R}\sum_{r\;=\;1}^{R}{\left(\partial J_{r}/\partial A^{p}\right)}_{\text{eq}}A^{p}A^{r}\leq 0$$

Equation (2) is a negative semidefinite quadratic form in affinities and the well-known conditions for a quadratic form to be negative semidefinite can be applied. These conditions give restrictions on rate coefficients (rate constants) in the thermodynamic polynomial; in other words, they put additional conditions on the thermodynamic consistency of rate equations. However, the transformation of the above function of concentrations, **J**(*T*, **c**), to the function of affinities is a mathematical procedure which requires correct and rigorous steps. These steps include expressing chemical potentials as functions of concentrations, resulting in the appearance of both chemical and so-called constitutive affinities (Pekař and Samohýl, 2014; Pekař, 2016). The results are generally **J**(*T*, **c**) = **J**(*T*, μ) = **J**(*T*, **A**, **B**), where the vector μ contains chemical potentials, the vector **A** contains chemical affinities, and the vector **B** contains constitutive affinities. Condition (2) is applied to the final function to find restrictions on rate parameters (constants).

## Overview of analyzed reacting systems

The method described in previous works (Pekař and Samohýl, 2014; Pekař, 2016) and outlined in the introduction is applied here on four different reacting mixtures in order to compare its outcomes for the resulting first and second order kinetic models. First, all four reacting systems are overviewed together with their models of different orders which result from (1). Then, condition (2) is applied on all models. Although in this method the order is a matter of the degree of the approximating polynomial in (1) and not a matter of *a priori* kinetic considerations, these two views are related. To demonstrate this is a further aim of this work. Beforehand, however, the relevant terminology should be clarified. Strictly speaking, degree refers to the polynomial in (1) and order to traditional rate equations. However, these two terms are closely connected, as will be seen in what follows. Polynomial degrees are whole numbers only and in a kinetic interpretation of the thermodynamic polynomial they correspond to orders—in this sense and according to this interpretation the two concepts can be used and should be understood interchangeably.

The first reacting system is a simple mixture of two isomers, A and B. Chemically, the simplest reaction here is assumed to be in the form:

(R1a) A ⇆ B,

which, as written, invokes first order kinetics. Its second order version can be expressed in traditional kinetics as

(R1b) 2A⇆2B.

In this mixture, only one independent reaction is possible; the vector **J** is thus one-dimensional. Reaction (R1a) is selected as the independent reaction and the second-degree thermodynamic polynomial can be written as (for derivation see Supplementary Material):

(3) $$\begin{array}{l} J=k_{10}(c_{\text{A}}-K^{-1}c_{\text{B}})\\ \;+\;k_{20}(c_{\text{A}}^{2}-K^{-1}c_{\text{A}}c_{\text{B}})+\;k_{02}(c_{\text{B}}^{2}-Kc_{\text{A}}c_{\text{B}}) \end{array}$$

where the vectors **k** are also one-dimensional and *K* is the thermodynamic equilibrium constant of the independent reaction. At the same time, the first term on the right hand side of (3) represents the first-degree thermodynamic polynomial.

The second analyzed system is composed of two single components, A and B, and their compound AB. Here, the combination reaction

(R2) A+B⇆AB

occurs naturally and is of the second order (forward direction). Also here, only one independent reaction exists—the most natural selection is just the reaction (R2). The first-degree thermodynamic polynomial vanishes in this reacting mixture; consequently, only the second-degree (or possibly higher) polynomial is reasonable. It has the following very simple form

(4) $$J=k_{110}(c_{A}c_{B}-K^{-1}c_{\text{AB}}),$$

which resembles the traditional mass-action expression; *K* is the thermodynamic equilibrium constant of the selected independent reaction (R2). Note that, here also, the vector **J** is one-dimensional. The vanishing first-degree thermodynamic polynomial is a natural consequence of the mathematical non-existence of paired pure first-power terms in the polynomial. However, this does not mean that kinetically first-order terms are missing—cf. the last term on the right hand side of (4).

The third system is just the mixture of three isomers (A, B, and C) analyzed in the previous work, but, here, modeled with the second degree thermodynamic polynomial:

(5) $$\begin{array}{l} J=k_{100}(c_{\text{A}}-K_{1}^{-1}c_{\text{B}})+k_{001}(c_{\text{C}}-K_{2}c_{\text{B}})+k_{020}(c_{\text{B}}^{2}-K_{1}^{2}c_{\text{A}}^{2})\\ \;+\;k_{002}(c_{\text{C}}^{2}-K_{1}^{2}K_{2}^{2}c_{\text{A}}^{2})+k_{110}(c_{\text{A}}c_{\text{B}}-K_{1}c_{\text{A}}^{2})+k_{101}(c_{\text{A}}c_{\text{C}}\\ \;-\;K_{1}K_{2}c_{\text{A}}^{2})+k_{011}(c_{\text{B}}c_{\text{C}}-K_{1}^{2}K_{2}c_{\text{A}}^{2}) \end{array}$$

Here, two independent reactions are possible and are selected (Pekař, 2016) as A = B and B = C with the corresponding thermodynamic equilibrium constants *K*_1_ and *K*_2_, resp. The vectors **J** and **k** are thus two-dimensional, the former containing the rates of the two independent reactions: **J** = (*J*_1_, *J*_2_). The first two terms on the right hand side of (5) represent the first-degree thermodynamic polynomial which was analyzed in the previous work (Pekař, 2016).

Traditionally, “triangular” reactions, (R3), are assumed to occur in this mixture, one of them being dependent.

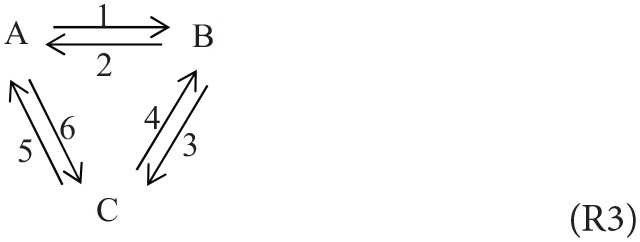



The last (fourth) system is similar and is that suggested by the reviewer of the previous work. Here, also, a compound of two isomers is included and the mixture thus contains the following components: A, B, AB, BA where (only) AB and BA are isomers. Analogically to (R3), a triangular scheme (R4) is suggested here in traditional kinetics.

The number of independent reactions is still two. The second-degree thermodynamic polynomial is:

(6) $$\begin{array}{l} J=k_{0001}(c_{\text{BA}}-K_{2}c_{\text{AB}})+k_{1100}(c_{\text{A}}c_{\text{B}}-K_{1}^{-1}c_{\text{AB}})\\ \;+\;k_{0002}(c_{\text{BA}}^{2}-K_{2}^{2}c_{\text{AB}}^{2})+k_{0011}(c_{\text{AB}}c_{\text{BA}}-K_{2}c_{\text{AB}}^{2})\\ \;+\;k_{1001}(c_{\text{A}}c_{\text{BA}}-K_{2}c_{\text{A}}c_{\text{AB}})+k_{0101}(c_{\text{B}}c_{\text{BA}}-K_{2}c_{\text{B}}c_{\text{AB}}) \end{array}$$

The vectors **J** and **k** are two-dimensional, the first term on the right hand side of (6) representing the only possible first-degree term. The two independent reactions are selected as A + B = AB and AB = BA with corresponding thermodynamic equilibrium constants *K*_1_ and *K*_2_, resp.

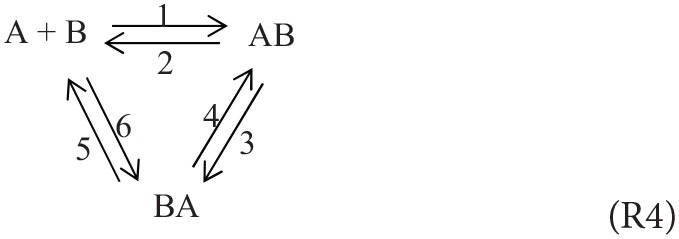



## Results and discussion

Now, the results of the thermodynamic analysis of these four reacting systems are discussed. Crucial for this analysis is the application of condition (2).

### Reacting system 1; a mixture of two isomers

The first-degree thermodynamic polynomial contains only one rate coefficient (also called here “rate constant”), namely *k*_10_ (the bold symbol is not retained due to the one-dimensionality of **k**_10_ from (3), similarly for other one-dimensional quantities). The transformed thermodynamic polynomial corresponding to the function **J**(*T*, **A**, **B**) is (for derivation see Supplementary Material):

(7) $$J=k_{10}\exp\frac{-\mu_{\text{A}}^{o}}{RT}\exp\frac{B}{RT}\exp\frac{A}{2RT}\left(\exp\frac{-A}{RT}-1\right)$$

where ° denotes the standard state and *A* and *B* are the two one-dimensional affinities. Condition (2) results here in the following simple expression:

(8) $${\left(\frac{\partial J}{\partial A}\right)}_{\text{eq}}=-(1/RT)k_{10}\exp\frac{-\mu_{\text{A}}^{o}}{RT}\exp\frac{B_{\text{eq}}}{RT}\leq 0$$

and from this it follows that *k*_10_ > 0 (*k*_10_ = 0 makes no sense in a reacting mixture), which is consistent with A being the reactant and with its component rate *J*^A^ being equal to –*J*, cf. also (3), where *J*^A^ represents reactant A's formation rate. Thus, the traditional mass-action kinetics expressed in the form of the first-degree version of (3), including the sign of the rate constant, is fully consistent with non-equilibrium thermodynamics—particularly, with entropic inequality (the second law). It should be noted that the presented method does not inherently include restrictions on the non-negativity of concentrations; these should be added as additional constraints.

In the case of the second-degree polynomial, the transformation to the function **J**(*T*, **A**, **B**) is more complex than (7) and can be found in Supplementary Material. Here, only the final restriction resulting from (2) is shown:

(9) $$k_{10}+k_{20}c_{\text{A},\text{eq}}-k_{02}K^{2}c_{\text{A},\text{eq}}\geq 0,$$

which should be fulfilled for an arbitrary equilibrium concentration. This enables the following theorem^1^ to be applied (the theorem was not necessary in the first-degree models).

**Theorem**. If the inequality

(10) a+bx≥0,

where *a, b, x* are real numbers, is valid for any positive *x*, then it is necessary and sufficient that

(11) a≥0, b≥0.

*Proof*. The sufficiency is obvious. The necessity is proven by contradiction. First, (10) is not fulfilled for any *x* > 0 if *a, b* are chosen as any of these three combinations: 1) *a* = 0, *b* < 0; 2) *a* < 0, *b* = 0; 3) *a* < 0, *b* < 0. If *a, b* are chosen as *a* > 0, *b* < 0 or *a* < 0, *b* > 0 then (10) is not fulfilled for any (positive) *x* < –*a*/*b* > 0. For all other combinations of *a* and *b*, i.e., those satisfying (11), a positive *x* not fulfilling (10) does not exist. Q.E.D.

Note that the theorem can be easily modified to *c*+*dx* ≤ 0 instead of (10); in this case, *c* ≤ 0, *d* ≤ 0. The theorem evidently also enables the condition of the non-negativity of concentrations to be included, albeit in an indirect way.

The theorem thus gives:

(12) $$k_{10}\geq 0$$

(13) $$k_{20}-k_{02}K^{2}\geq 0$$

The first condition (12) is the same as that found above for the first-degree polynomial. Thus, both polynomials give consistent conditions for the first order rate constant (*k*_10_). Referring to terminology introduced in a previous work (Pekař, 2016), the coefficient *k*_10_ is a proper coefficient, while *k*_20_ and *k*_02_ are examples of coupling coefficients. However, in this case, the coupling coefficients are of a somewhat different type than in Pekař (2016), where they coupled the selected independent reactions in their rate equations. Here, they couple the rate of the independent reaction with expressions which could be viewed as rates of additional, dependent, reactions, cf. (3).

Let us further suppose that

(14) $$k_{20}\geq 0$$

Then, condition (13) is fulfilled (also) for $k_{02}=-K^{-2}k_{20},$ in which case $k_{20}+k_{02}K^{2}=0$. Consequently, reaction rate (3) is transformed into the following expression:

(15) $$J=k_{10}(c_{\text{A}}-K^{-1}c_{\text{B}})+k_{20}(c_{\text{A}}^{2}-K^{-2}c_{\text{B}}^{2})$$

This, with inequality symbols only in (12) and (14), gives the traditional expression for *J*^A^ (i.e., *J*^A^ = –*J*) when (R1a) and (R1b) occur simultaneously. Thus, this traditional expression is achieved as a result of necessary condition (12) and a sufficient version of condition (13), and, as such, this special case is thus compatible with our thermodynamic requirements.

### Reacting system 2; a mixture of three components, one of them being compound of the other two

As stated above, the first degree thermodynamic polynomial vanishes here. The second degree polynomial in terms of affinities is as follows (for derivation see Supplementary Material):

(16) $$J=k_{110}\exp\frac{-\mu_{\text{A}}^{o}-\mu_{\text{B}}^{o}}{RT}\exp\frac{B^{1}+B^{2}}{RT}\exp\frac{A}{3RT}\left(\exp\frac{-A}{RT}-1\right)$$

Condition (2) is detailed in Supplementary Material; its main result is that *k*_110_ ≥ 0, which is fully consistent with the fact that, for example, *J*^A^ = −*J* (remember the negative stoichiometric coefficients of reactants), where *J*^A^ represents reactant A's formation rate and *J* is the rate of (the selected independent) reaction R2, cf. (4).

Because the zero rate constant is impossible for A really reacting with B, the positiveness of the rate constant, traditionally supposed in mass-action kinetics, is shown here to be a condition for consistency with thermodynamics (the second law).

In this example, the thermodynamic polynomial contains only (one) proper coefficient, cf. (4).

### Reacting system 3; a mixture of three isomers

Our previous work (Pekař, 2016) also compared traditional, first-order, mass-action rate equations with those given by the thermodynamic polynomial of the first degree. Therefore, we start the discussion of this example by similar comparison in the case of the second order (degree). The first order triangular scheme (R3) is added with its second order analog, i.e., scheme (R3) with all stoichiometric coefficients equal to two. The mass-action rate equations in a batch system are as follows:

$$r_{11}=k_{1}c_{\text{A}}-k_{2}c_{\text{B}},\;r_{12}=k_{7}c_{\text{A}}^{2}-k_{8}c_{\text{B}}^{2}$$

$$r_{21}=k_{3}c_{\text{B}}-k_{4}c_{\text{C}},\;r_{22}=k_{9}c_{\text{B}}^{2}-k_{10}c_{\text{C}}^{2}$$

(17) $$r_{31}=k_{5}c_{\text{C}}-k_{6}c_{\text{A}},\;r_{32}=k_{11}c_{\text{C}}^{2}-k_{12}c_{\text{A}}^{2}$$

(18a) $$\text{d}c_{\text{A}}/\text{d}t\;=-r_{11}+r_{31}-r_{12}+r_{32}$$

(18b) $$\text{d}c_{\text{B}}/\text{d}t\;=r_{11}-r_{21}+r_{12}-r_{22}$$

(18c) $$\text{d}c_{\text{C}}/\text{d}t\;=r_{21}-r_{31}+r_{22}-r_{32}$$

The relationship between the traditional rates and the rates based on our thermodynamic approach are (for details see Supplementary Material):

(19a) $$J_{1}=r_{11}-r_{31}+r_{12}-r_{32}$$

(19b) $$J_{2}=r_{21}-r_{31}+r_{22}-r_{32}$$

which is more complex than without the second order (degree), cf. Pekař (2016), as expected. However, the second order rates are mathematically included in the independent reaction rates in the same way as their first order counterparts.

To be able to find restrictions on the traditional rate constants, the thermodynamic polynomial (5) should retain only those second degree terms which correspond to the reactions between identical isomers. In other words, we select **k**_110_ = **k**_101_ = **k**_011_ = **0**.Using the same procedure as previously (Pekař, 2016), we find the same relationships between first order rate constants and first degree polynomial coefficients. In the case of the second order (degree), the following identities are found:

(20) $$k_{8}=-k_{020}^{1},\;k_{9}=k_{020}^{2},\;k_{11}=-k_{002}^{1}$$

together with the additional restrictions:

(21) $$k_{10}=k_{002}^{1}-k_{002}^{2}$$

(22) $$k_{7}+k_{12}=-k_{020}^{1}K_{1}-k_{002}^{1}K_{1}^{2}K_{2}^{2}$$

(note that, for example, $k_{020}^{1}$ refers to the first independent reaction, whereas $k_{020}^{2}$ refers to the second one). Thus, out of six second order traditional rate constants, only four are independent and determine the values of the remaining two; for example:

(23) $$k_{7}=(k_{8}+k_{9})K_{1}-k_{10}K_{1}^{2}K_{2}^{2}$$

(24) $$k_{12}=-k_{9}K_{1}+(k_{10}+k_{11})K_{1}^{2}K_{2}^{2}$$

This is similar to the first order scheme (R3), where two first order rate constants were also not independent (Pekař, 2016). Further, if equilibrium is defined by *r*_*ij*_ = 0, then from *r*_*i*__2_ in (17) it is very easy to derive an additional, “detailed balance” condition for the second order rate constants:

(25) $$k_{7}k_{9}k_{11}=k_{8}k_{10}k_{12}$$

which, exactly as in the case of the first order (Pekař, 2016), decreases the number of independent second order rate constants to three.

The transformation of (5) into the function of affinities is very complex in this example, and can be found in Supplementary Material as well as the results of applying condition (2) to the full thermodynamic polynomial, which are rather complex and general. We therefore restrict our discussion to a simplified version which contains only those second order terms in the thermodynamic polynomial which correspond to reactions between identical isomers as above; for example, 2 A = 2 B. In other words, we set **k**_110_ = **k**_101_ = **k**_011_ = **0**, again, and reactions such as A + B = 2 C are not considered.

With this modification, condition (2) leads to restrictions on two (proper) first order rate constants which are fully consistent with those obtained previously with the first-order polynomial (Pekař, 2016):

(26) $$k_{100}^{1}\geq 0,\;k_{001}^{2}\leq 0.$$

Further, the following explicit restrictions for three second order constants can be derived from (2):

(27) $$k_{002}^{1}\leq 0,\;k_{020}^{1}\leq 0,\;k_{002}^{2}\leq 0.$$

All three are coupling constants but the latter two couple an independent reaction with its double. There are no similar simple restrictions on the remaining three rate constants which are of a coupling type (coupling with rates of reactions which do not belong to the selected independent reactions). They should conform to a more complex condition given in Supplementary Material.

### Reacting system 4; a mixture of four components capable of isomerization and combination reactions

Note that the first term on the right hand side of (6) represents the (only) first order contribution and corresponds to the reaction AB = BA (one of the two selected independent reactions). If only the first degree thermodynamic polynomial is considered, condition (2) gives the following restrictions (see Supplementary Material for details):

(28) $$k_{0001}^{1}=0,\;k_{0001}^{2}\leq 0.$$

This means that *J*_1_ = 0, which is consistent with the fact that the first selected independent reaction (A+B = AB) is of the second order. Thus, our analysis independently underlines the fact that the whole triangular scheme (R4) cannot be modeled solely by a first order kinetic model. The second (inequality) condition, which in real reactions is $k_{0001}^{2}<0,$ is consistent with the fact that *J*^AB^ = –*J*_2_.

All terms in (6) formally form the following reaction scheme (Pekař, 2009):

1. AB = BA,

2. A + B = AB,

3. 2 AB = 2 BA,

4. 2 AB = AB + BA,

5. A + AB = A + BA,

6. B + AB = B + BA.

Only the first two reactions directly reflect what chemists would expect for the triangular scheme (R4) (the third reaction in this triangle is not independent). Therefore, similarly as in the preceding triangular example, we will restrict our discussion to the simplified polynomial. This also enables comparison with traditional mass-action kinetics. Thus, we put

$$k_{0002}=k_{0011}=k_{1001}=k_{0101}=0.$$

The vector of reaction rates expressed as a function of affinities is then:

(29) $$\begin{array}{l} \;J=k_{0001}\exp\frac{-\mu_{\text{BA}}^{o}}{RT}\exp\frac{B_{1}+B_{2}}{RT}\exp\frac{A^{1}-2A^{2}}{5RT}\left(\exp\frac{A^{2}}{RT}-1\right)\\ +\;k_{1100}\exp\frac{-\mu_{\text{A}}^{o}-\mu_{\text{B}}^{o}}{RT}\exp\frac{B_{1}+B_{2}}{5RT}\exp\frac{A^{1}-2A^{2}}{5RT}\left(\exp\frac{-A^{1}}{RT}-1\right) \end{array}$$

Condition (2) then gives the following simple and explicit restrictions (see Supplementary Material for details):

(30) $$k_{0001}^{2}\leq 0;\;k_{1100}^{1}\geq 0.$$

Both constants in (30) are of the proper type. The conditions in (30) are consistent with the fact that *J*^AB^ = *J*_1_ − *J*_2_. The first condition in (30) also corresponds to the second condition in (28) found for the first order model. The restrictions on the remaining two (coupling) rate constants are not as simple, again. They should satisfy the following condition (see Supplementary Material for details):

(31) $${\left(\frac{1}{2}k_{1100}^{2}-\frac{1}{2}k_{0001}^{1}K_{1}K_{2}\right)}^{2}\leq -k_{0001}^{2}k_{1100}^{1}K_{1}K_{2}.$$

Note, that due to (30), the right hand side of (31) is positive, as required.

Also in this case we can derive additional constraints on the rate constants of the traditional mass-action kinetic model of scheme (R4)—see Supplementary Material for details on this procedure. The result is that the values of at least two traditional rate constants are determined by the values of the remaining four constants (together with the equilibrium constants). For instance:

(32) $$k_{2}=-k_{5}K_{2}+(k_{1}+k_{6})\;K_{1}^{-1},\;k_{3}=(k_{4}+k_{5})K_{2}-k_{6}K_{1}^{-1}.$$

(the numbering of the rate constants corresponds to the numbers shown in scheme (R4)). Thus, the dimensionality of parameter estimation problems is reduced by two.

It can also be easily verified that the traditional mass-action rate equations of scheme (R4) also lead to a ”detailed balance” constraint on their rate constants, very similar to that of the “first order” triangular scheme (R3). This is another dimensionality reduction. The number of independent rate constants is therefore three, the same as in the case of the first order scheme (R3) analyzed in our previous work (Pekař, 2016).

## Conclusions

Second-order kinetic models—or, more specifically, second-degree thermodynamic polynomials—further demonstrated the power of the presented thermodynamic analysis and also revealed where simple, specific results (restrictions) cannot be expected. Second-degree polynomial models led to more complex results arising from thermodynamic condition (2), but in all cases, regardless of the degree (order) and overlooking some of the simplifications made above, simple sign restrictions were found for proper rate constants (coefficients). In contrast, restrictions on coupling constants were usually weaker, giving no strict restrictions on their sign, thus allowing them more “freedom.” This means more freedom in kinetic coupling between different reactions forming a reaction scheme, i.e., in the kinetic effects of other reactions on the rate of some reaction in a reacting system; at the same time, zero coupling (zero coupling rate constants) is not excluded by these final thermodynamic restrictions.

The comparison of thermodynamic polynomials with traditional mass-action rate models revealed constraints on the rate constants of the latter which could not be revealed using the traditional approach. The thermodynamic methodology presented in this work thus allows a reduction in the number of kinetic parameters to be evaluated from data, i.e., a reduction in the dimensionality of the parameter estimation problem.

In this analysis, the universality and consistency of the presented method was thus further underlined. It not only allows rate equations to be derived on the basis of results of non-equilibrium thermodynamics but also enables the derivation of constraints on their rate coefficients which are necessary for full consistency with entropy inequality (the second law of thermodynamics). To this end, the proper transformation of rate equations to functions of chemical and constitutive affinities should be performed.

## Author contributions

The author confirms being the sole contributor of this work and approved it for publication.

### Conflict of interest statement

The author declares that the research was conducted in the absence of any commercial or financial relationships that could be construed as a potential conflict of interest.

## Footnotes

^1^The theorem and its proof are provided by Vít Samohýl.
