# Supplementary material for: Thermodynamic Analysis of Chemically Reacting Mixtures—Comparison of First and Second Order Models
Source: Front Chem. 2018 Mar 1;6:35. doi: 10.3389/fchem.2018.00035 (PMC5838023; doi:10.3389/fchem.2018.00035)
Supplement: Supplementary file 1 [file DataSheet1.DOCX]

Supplementary Material

Thermodynamic analysis of chemically reacting mixtures – comparison of first and second order models

Miloslav Pekař *

*** Correspondence:** Corresponding Author: pekar@fch.vut.cz

**Note on equation numbering.** Equations which also appear in the main text retain their main text number here.

**General note.** The basics of the method are explained in Pekař and Samohýl (2014); further details can be found in previous work by Pekař (2009, 2010, 2016).

**1. Simple isomerisation.**

**1.1. Equation (3)**. The full second degree thermodynamic polynomial is as follows (the vector **J** is one-dimensional; bold symbols are not retained here for one-dimensional quantities):

|  | $J=k_{00}+k_{10}c_{A}+k_{01}c_{B}+k_{20}c_{A}^{2}+k_{02}c_{B}^{2}+k_{11}c_{A}c_{B}.$ | (S1) |
| --- | --- | --- |

In equilibrium, defined as $J=0,$ we have, after substitution from the equality $c_{B,eq}=Kc_{A,eq}$(which follows from the expression for the equilibrium constant of the independent reaction):

|  | $0=k_{00}+(k_{10}+k_{01}K)c_{A,eq}+(k_{20}+k_{02}K^{2}+k_{11}K)c_{A,eq}^{2}.$ | (S2) |
| --- | --- | --- |

Because (S2) should be valid for any equilibrium concentration, it follows that $k_{00}=0,$ $\left( k_{10}+k_{01}K \right)=0,$ and $\left( k_{20}+k_{02}K^{2}+k_{11}K \right)=0$. Using these identities, the final version of the thermodynamic polynomial is as follows:

|  | $J=k_{10}(c_{A}-K^{-1}c_{B})+k_{20}(c_{A}^{2}-K^{-1}c_{A}c_{B})+k_{02}(c_{B}^{2}-Kc_{A}c_{B}).$ | (3) |
| --- | --- | --- |

An analogical procedure was also used to derive the other thermodynamic polynomials given in the main text, i.e., (4)-(6).

**1.2. Equation (7)**. The transformation to the function of affinities is based on the procedure which can be found in Pekař and Samohýl (2014)^[[1]](#footnote-1)^. The composition matrix (Pekař and Samohýl, 2014; p.150) is $\left\| T_{\sigma\alpha} \right\|=\left[ \begin{matrix} 1 & 1 \end{matrix} \right]\equiv\left\| S_{\sigma\alpha} \right\|;\sigma=1;\alpha=1, 2;$ the stoichiometric matrix of the selected independent reaction (Pekař and Samohýl, 2014; p.153) is $\left\| P^{p\alpha} \right\|=\left[ \begin{matrix} -1 & 1 \end{matrix} \right];p=1$ (A = 1, B = 2). The only basis vector $\vec{f}$ (Pekař and Samohýl, 2014; p.152) is $\vec{f}_{1}=\vec{e}^{1}+\vec{e}^{2}.$ The only basis vector $\vec{g}$ (Pekař and Samohýl, 2014; p.153) is $\vec{g}^{1}=-\vec{e}_{1}+\vec{e}_{2}$ and the two metric tensors (Pekař and Samohýl, 2014; p.154) are:

|  | $g^{11}=\vec{g}^{1}.\vec{g}^{1}=2; g_{11}=\frac{1}{g^{11}}=\frac{1}{2}.$ | (S3) |
| --- | --- | --- |

The relationship between chemical potential and affinities (Pekař and Samohýl, 2014; p.182) is as follows:

|  | $\mu_{\alpha}=A^{1}g_{11}P^{1\alpha}+B^{1}S_{1\alpha}.$ | (S4) |
| --- | --- | --- |

where $A^{1}$ is the chemical affinity (henceforth denoted $A$) and $B^{1}$ is the constitutive affinity (henceforth denoted $B$) . Thus:

|  | $\mu_{1}=-\frac{1}{2}A+B; \mu_{2}=\frac{1}{2}A+B.$ | (S5) |
| --- | --- | --- |

Further (Pekař and Samohýl, 2014; p.181):

|  | $A=\mu_{1}P^{11}+\mu_{2}P^{12}=-\mu_{1}+\mu_{2}$ | (S6) |
| --- | --- | --- |

and from (S5):

|  | $B=\frac{1}{2}\mu_{1}+\frac{1}{2}\mu_{2}.$ | (S7) |
| --- | --- | --- |

An ideal chemical potential is supposed, i.e. $\mu_{\alpha}=\mu_{\alpha}^{o}+RT \ln c_{\alpha}.$ In equilibrium, $A=0$ and from (S6)

|  | $\mu_{1}^{o}-\mu_{2}^{o}=RT \ln K, K={c_{2,\mathrm{eq}}}/{c_{1,\mathrm{eq}}}.$ | (S8) |
| --- | --- | --- |

Substituting $c_{\alpha}=\exp[\left( \mu_{\alpha}-\mu_{\alpha}^{o} \right)/{RT}]$ and $K$ from the first equality of (S8) into (3) with the first degree term only and using (S5)-(S8), equation (7) is obtained.

**1.3. Second degree thermodynamic polynomial in affinities and equation (9)**. Equation (3) can be rewritten as

|  | $J={(k}_{10}+k_{20}c_{A}-k_{02}Kc_{B})(c_{A}-K^{-1}c_{B}).$ | (S9) |
| --- | --- | --- |

The substitution of chemical potentials for concentrations and equilibrium constant (see (S8)) gives

|  | $J=\left[ k_{10}+k_{20}\exp\left( \frac{\mu_{A}-\mu_{A}^{o}}{RT} \right)-k_{02}\exp\left( \frac{\mu_{A}^{o}-\mu_{B}^{o}}{RT} \right)\exp\left( \frac{\mu_{B}-\mu_{B}^{o}}{RT} \right) \right]$  $\left[ \exp\left( \frac{\mu_{A}-\mu_{A}^{o}}{RT} \right)-\exp\left( \frac{\mu_{B}^{o}-\mu_{A}^{o}}{RT} \right)\exp\left( \frac{\mu_{B}-\mu_{B}^{o}}{RT} \right) \right].$ | (S10) |
| --- | --- | --- |

Substituting from (S5) and after some algebraic manipulation, the final function **J**(*T*, **A**, **B**) is obtained:

|  | $J=\left[ E_{1}+E_{2}\exp\left( \frac{-A}{2RT} \right)-E_{3}\exp\left( \frac{A}{2RT} \right) \right]E_{4}\exp\left( \frac{A}{2RT} \right)\left[ \exp\left( \frac{-A}{RT} \right)-1 \right]$ | (S11) |
| --- | --- | --- |

where

|  | $E_{1}=k_{10}\exp\left( \frac{-\mu_{A}^{o}}{RT} \right),$ | (S12a) |
| --- | --- | --- |
|  | $E_{2}=k_{20}\exp\left( \frac{-2\mu_{A}^{o}}{RT} \right)\exp\left( \frac{B}{RT} \right),$ | (S12b) |
|  | $E_{3}=k_{02}\exp\left( \frac{-2\mu_{B}^{o}}{RT} \right)\exp\left( \frac{B}{RT} \right),$ | (S12c) |
|  | $E_{4}=\exp\left( \frac{B}{RT} \right).$ | (S12d) |

Note, that in equilibrium, where $A=0$, $J=0$ from (S11), as expected.

The derivative $\partial J/\partial A$ is calculated from (S11) and its equilibrium value inserted into (2); the corresponding quadratic form condition is then:

|  | $-{[\left( E_{1}+E_{2}-E_{3} \right)E_{4}]}_{\mathrm{eq}}/RT\leq0.$ | (S13) |
| --- | --- | --- |

Only the (equilibrium) constitutive affinity remains in (S13) and it can be back-substituted using (S7):

|  | $B_{\mathrm{eq}}=\frac{1}{2}\mu_{1,eq}+\frac{1}{2}\mu_{2,eq}=\frac{1}{2}\left( \mu_{A}^{o}+\mu_{B}^{o} \right)+\frac{1}{2}RT\ln c_{A,\mathrm{eq}}c_{B,\mathrm{eq}}.$ | (S14) |
| --- | --- | --- |

The result is

|  | $k_{10}+\left[ k_{20}\exp\left( \frac{-\mu_{A}^{o}+\mu_{B}^{o}}{2RT} \right)-k_{02}\exp\left( \frac{3\mu_{A}^{o}-3\mu_{B}^{o}}{2RT} \right) \right]\left( c_{A,\mathrm{eq}}c_{B,\mathrm{eq}} \right)^{\frac{1}{2}}\geq0.$ | (S15) |
| --- | --- | --- |

When the equilibrium constant is substituted from both parts of (S8), equation (9) follows.

**2. Combination reaction**. Here, the only basis vector $\vec{g}$ (Pekař and Samohýl, 2014; p.153) is $\vec{g}^{1}=-\vec{e}_{1}-\vec{e}_{2}+\vec{e}_{3}$ and the two metric tensors (Pekař and Samohýl, 2014; p.154) are:

|  | $g^{11}=\vec{g}^{1}.\vec{g}^{1}=3; g_{11}=\frac{1}{g^{11}}=\frac{1}{3}.$ | (S16) |
| --- | --- | --- |

The relationship between chemical potential and affinities (Pekař and Samohýl, 2014; p.182) are here:

|  | $\mu_{\alpha}=A^{1}g_{11}P^{1\alpha}+B^{1}S_{1\alpha}+B^{2}S_{2\alpha}.$ | (S17) |
| --- | --- | --- |

where the matrix $\left\| S_{\sigma\alpha} \right\|;\sigma=1, 2;\alpha=1, 2, 3$ is identical with the composition matrix (Pekař and Samohýl, 2014; p.150):

|  | $\left\Vert S_{\sigma\alpha} \right\Vert\equiv\left\Vert T_{\sigma\alpha} \right\Vert=\left[ \begin{matrix} 1 & 0 & 1 \\ 0 & 1 & 1 \end{matrix} \right]$ | (S18) |
| --- | --- | --- |

(A = 1, B = 2, AB = 3) and the stoichiometric matrix of the selected independent reaction (Pekař and Samohýl, 2014; p.153) is $\left\| P^{p\alpha} \right\|=\left[ \begin{matrix} -1 & -1 & 1 \end{matrix} \right];p=1.$ The relationship between chemical affinity and chemical potentials (Pekař and Samohýl, 2014; p.181) is:

|  | $A^{1}=\mu_{1}P^{11}+\mu_{2}P^{12}+\mu_{3}P^{13}=-\mu_{1}-\mu_{2}+\mu_{3}.$ | (S19) |
| --- | --- | --- |

Equation (S17) gives

|  | $\mu_{A}=-\frac{1}{3}A^{1}+B^{1}; \mu_{B}=-\frac{1}{3}A^{1}+B^{2}; \mu_{\mathrm{AB}}=\frac{1}{3}A^{1}+B^{1}+B^{2}.$ | (S20) |
| --- | --- | --- |

The two constitutional affinities are then expressed in terms of chemical potentials as follows:

|  | $B^{1}=\frac{2}{3}\mu_{A}-\frac{1}{3}\mu_{B}+\frac{1}{3}\mu_{\mathrm{AB}},$ | | (S21a) |
| --- | --- | --- | --- |
|  | $B^{2}=-\frac{1}{3}\mu_{A}+\frac{2}{3}\mu_{B}+\frac{1}{3}\mu_{\mathrm{AB}}.$ | (S21b) | |

Further,

|  | $-RT \ln K=-\mu_{A}^{o}-\mu_{B}^{o}+\mu_{\mathrm{AB}}^{o}, K={c_{\mathrm{AB},\mathrm{eq}}}/{(c_{A,\mathrm{eq}}}c_{B,\mathrm{eq}}).$ | (S22) |
| --- | --- | --- |

The substitution of chemical potentials into (4), which was derived by a similar procedure to that shown above under 1.1, gives

|  | $J=k_{110}\exp\left( \frac{-\mu_{A}^{o}-\mu_{B}^{o}}{RT} \right)\left[ \exp\left( \frac{\mu_{A}+\mu_{B}}{RT} \right)-\exp\left( \frac{\mu_{\mathrm{AB}}}{RT} \right) \right].$ | (S23) |
| --- | --- | --- |

Introducing affinities from (S19) and (S21) results in

|  | $J=k_{110}\exp\left( \frac{-\mu_{A}^{o}-\mu_{B}^{o}}{RT} \right)\exp\left( \frac{B^{1}+B^{2}}{RT} \right)\exp\left( \frac{A^{1}}{3RT} \right)\left[ \exp\left( \frac{-A^{1}}{RT} \right)-1 \right]$ | (S24) |
| --- | --- | --- |

which is equation (16). The equilibrium derivative in condition (2) is

|  | $\left( \frac{\partial J}{\partial A^{1}} \right)_{\mathrm{eq}}=k_{110}\exp\left( \frac{-\mu_{A}^{o}-\mu_{B}^{o}}{RT} \right)\exp\left( \frac{B_{\mathrm{eq}}^{1}+B_{\mathrm{eq}}^{2}}{RT} \right)\left( -\frac{1}{RT} \right).$ | (S25) |
| --- | --- | --- |

Because the equilibrium derivative should be smaller than or equal to zero to fulfill condition (2), it follows that $k_{110}\geq0$.

**3. Mixture of three isomers.**

**3.1. Equation (19)**. Component and reaction rates in a traditional mass-action approach for a second order system are related by

|  | $\left[ \begin{matrix} J^{A} & J^{B} & J^{C} \end{matrix} \right]=\left[ \begin{matrix} r_{11} & r_{21} & \end{matrix}\begin{matrix} r_{31} & r_{12} & \end{matrix}\begin{matrix} r_{22} & r_{32} \end{matrix} \right]\left[ \begin{aligned} \begin{matrix} -1 & 1 & 0 \\ 0 & -1 & 1 \\ 1 & 0 & -1 \end{matrix} \\ \begin{matrix} -1 & 1 & 0 \\ 0 & -1 & 1 \\ -1 & 0 & -1 \end{matrix} \end{aligned} \right]$ | (S26) |
| --- | --- | --- |

whereas in our thermodynamic approach they are related by

|  | $\left[ \begin{matrix} J^{A} & J^{B} & J^{C} \end{matrix} \right]=\left[ \begin{matrix} J_{1} & J_{2} \end{matrix} \right]\left[ \begin{matrix} -1 & 1 & 0 \\ 0 & -1 & 1 \end{matrix} \right].$ | (S27) |
| --- | --- | --- |

Comparing the right hand sides of (S26) and (S27) leads to equation (19).

**3.2. Second degree thermodynamic polynomial in affinities**. All relationships required to make the transformation to the function of affinities are the same as in my previous work (Pekař, 2016); they are listed here without comments:

|  | $A^{1}=-\mu_{A}+\mu_{B}; A^{2} =-\mu_{B}+\mu_{C}; B_{1}=\mu_{A}+\mu_{B}+\mu_{C}.$ | (S28) |
| --- | --- | --- |
|  | $B^{1}=B_{1}/3=(\mu_{A}+\mu_{B}+\mu_{C})/3.$ | (S29) |
|  | $\mu_{A}=\frac{-2A^{1}-A^{2}}{3}+B^{1}; \mu_{B}=\frac{A^{1}-A^{2}}{3}+B^{1}; \mu_{C}=\frac{A^{1}+2A^{2}}{3}+B^{1}.$ | (S30) |
|  | $K_{1}=\frac{c_{B,eq}}{c_{A,eq}}=\exp\left( \frac{\mu_{A}^{o}-\mu_{B}^{o}}{RT} \right); K_{2}=\frac{c_{C,eq}}{c_{B,eq}}=\exp\left( \frac{\mu_{B}^{o}-\mu_{C}^{o}}{RT} \right).$ | (S31) |

The transformation of first degree terms in thermodynamic polynomial (5), which was derived by a similar procedure to that shown above under 1.1, to the function of affinities can be found in the previous work (Pekař, 2016). Here we list the transformations of second degree terms, in which (S28)-(S31) were used:

|  | $k_{020}\left( c_{B}^{2}-K_{1}^{2}c_{A}^{2} \right)=$  $k_{020}\exp\left( \frac{-2\mu_{B}^{o}}{RT} \right)\exp\left( \frac{2}{3}\frac{A^{1}-A^{2}}{RT} \right)\exp\left( \frac{2B^{1}}{RT} \right)\left[ 1-\exp\left( \frac{-2A^{1}}{RT} \right) \right],$ | (S32) |
| --- | --- | --- |
|  | $k_{002}\left( c_{C}^{2}-K_{1}^{2}K_{2}^{2}c_{A}^{2} \right)=$  $k_{002}\exp\left( \frac{-2\mu_{C}^{o}}{RT} \right)\exp\left( \frac{2}{3}\frac{A^{1}+2A^{2}}{RT} \right)\exp\left( \frac{2B^{1}}{RT} \right)\left[ 1-\exp\left( 2\frac{-A^{1}-A^{2}}{RT} \right) \right],$ | (S33) |
|  | $k_{110}\left( c_{A}c_{B}-K_{1}c_{A}^{2} \right)=$  $k_{110}\exp\left( \frac{-\mu_{A}^{o}-\mu_{B}^{o}}{RT} \right)\exp\left( \frac{2}{3}\frac{{-2A}^{1}-A^{2}}{RT} \right)\exp\left( \frac{2B^{1}}{RT} \right)\left[ \exp\left( \frac{A^{1}}{RT} \right)-1 \right],$ | (S34) |
|  | $k_{101}\left( c_{A}c_{C}-K_{1}K_{2}c_{A}^{2} \right)=$  $k_{101}\exp\left( \frac{-\mu_{A}^{o}-\mu_{C}^{o}}{RT} \right)\exp\left( \frac{2}{3}\frac{{-2A}^{1}-A^{2}}{RT} \right)\exp\left( \frac{2B^{1}}{RT} \right)\left[ \exp\left( \frac{A^{1}+A^{2}}{RT} \right)-1 \right],$ | (S35) |
|  | $k_{011}\left( c_{B}c_{C}-K_{1}^{2}K_{2}c_{A}^{2} \right)=$  $k_{011}\exp\left( \frac{-\mu_{B}^{o}-\mu_{C}^{o}}{RT} \right)\exp\left( \frac{2}{3}\frac{{-2A}^{1}-A^{2}}{RT} \right)\exp\left( \frac{2B^{1}}{RT} \right)\left[ \exp\left( \frac{{2A}^{1}+A^{2}}{RT} \right)-1 \right].$ | (S36) |

All (first and second degree) terms are substituted into (5) and the function **J**(*T*, **A**, **B**) follows, which is not derived here (the derivation is left as a simple exercise for interested readers).

**3.3. Application of condition (2).** To apply this condition, the derivatives ${\partial\mathbf{J}}/{\partial A^{1}}$ and ${\partial\mathbf{J}}/{\partial A^{2}}$ should be calculated. Then, the requirements for the negative semidefiniteness of form (2) should be fulfilled:

|  | $\left( {\partial J_{1}}/{\partial A^{1}} \right)_{\mathrm{eq}}\leq0;\left( {\partial J_{2}}/{\partial A^{2}} \right)_{\mathrm{eq}}\leq0$ | (S37a) |
| --- | --- | --- |

and

|  | $\left[ ({\partial J_{1}}/{\partial A^{1})}\left( {\partial J_{2}}/{\partial A^{2}} \right)-\left( {\partial J_{2}}/{\partial A^{1}}+{\partial J_{1}}/{\partial A^{2}} \right)^{2}/4 \right]_{\mathrm{eq}}\geq0.$ | (S37b) |
| --- | --- | --- |

The two derivatives are:

|  | $\left( \frac{\partial\mathbf{J}}{\partial A^{1}} \right)_{\mathrm{eq}}=-\mathbf{k}_{100}X+\mathbf{k}_{020}2Z^{2}RT+\mathbf{k}_{002}2Y^{2}RT+\mathbf{k}_{110}XZRT+$  $\mathbf{k}_{101}XYRT+\mathbf{k}_{011}2YZRT,$ | (S38) |
| --- | --- | --- |
|  | $\left( \frac{\partial\mathbf{J}}{\partial A^{2}} \right)_{\mathrm{eq}}=\mathbf{k}_{001}Y+\mathbf{k}_{002}Y^{2}RT+\mathbf{k}_{101}XYRT+\mathbf{k}_{011}YZRT$ | (S39) |

where

|  | ${X=\frac{1}{RT}\left[ \exp\left( \frac{B^{1}-\mu_{A}^{o}}{RT} \right) \right]}_{\mathrm{eq}}=\frac{c_{A,eq}}{RT},$ | (S40) |
| --- | --- | --- |
|  | ${Y=\frac{1}{RT}\left[ \exp\left( \frac{B^{1}-\mu_{C}^{o}}{RT} \right) \right]}_{\mathrm{eq}}=\frac{c_{C,eq}}{RT},$ | (S41) |
|  | ${Z=\frac{1}{RT}\left[ \exp\left( \frac{B^{1}-\mu_{B}^{o}}{RT} \right) \right]}_{\mathrm{eq}}=\frac{c_{B,eq}}{RT},$ | (S42) |

In the derivation of (S40)-(S42), identities (S29) and (S31) were used.

The full conditions (S37) with (S38)-(S39) are very complex and are not further explored; note, however, that from (S37a) and (S39) it follows, for example, that all $\mathbf{k}_{ijk}$ in (S37b) cannot be positive. Only a simplification is discussed in the main text, for which

|  | $\left( \frac{\partial\mathbf{J}}{\partial A^{1}} \right)_{\mathrm{eq}}=-\mathbf{k}_{100}X+\mathbf{k}_{020}2Z^{2}RT+\mathbf{k}_{002}2Y^{2}RT,$ | (S43) |
| --- | --- | --- |
|  | $\left( \frac{\partial\mathbf{J}}{\partial A^{2}} \right)_{\mathrm{eq}}=\mathbf{k}_{001}Y+\mathbf{k}_{002}Y^{2}RT.$ | (S44) |

Then, (S44) and (S37a) give

|  | $k_{001}^{2}+k_{002}^{2}c_{C,eq}\leq0.$ | (S45) |
| --- | --- | --- |

Applying the theorem stated in the main text, we have the corresponding parts of (26) and (27). Further, (S43) and (S37a) give

|  | $\left( -k_{100}^{1}+2K_{1}^{2}k_{020}^{1}c_{A,eq} \right)c_{A,eq}+2k_{002}^{1}c_{C,eq}^{2}\leq0.$ | (S46) |
| --- | --- | --- |

Selecting a fixed but arbitrary value for $c_{A,eq}$, it follows from the same theorem that $k_{002}^{1}\leq0,$ which is a part of (27), and that $-k_{100}^{1}+2K_{1}^{2}k_{020}^{1}c_{A,eq}\leq0;$ applying the theorem to the latter inequality, the remaining parts of (26) and (27) follow.

Condition (S37b) leads, in the simplified case, to

|  | $W-\left[ \left( -k_{100}^{2}+2K_{1}^{2}k_{020}^{2}c_{A,eq} \right)c_{A,eq}+2k_{002}^{2}c_{C,eq}^{2}+(k_{001}^{1}+k_{002}^{1}c_{C,eq})c_{C,eq} \right]^{2}\geq0$ | (S47) |
| --- | --- | --- |

where $W\geq0$ contains only “already restricted”(by (26) or (27)) rate coefficients; (S47) is a rather complex condition which should be fulfilled by the remaining rate coefficients.

**4. Isomerisation and combination.**

**4.1. Transformation to the affinities function.** The matrix $\left\| S_{\sigma\alpha} \right\|;\sigma=1, 2;\alpha=1, \ldots, 4$ is identical with the composition matrix (Pekař and Samohýl, 2014; p.150):

|  | $\left\Vert S_{\sigma\alpha} \right\Vert\equiv\left\Vert T_{\sigma\alpha} \right\Vert=\left[ \begin{matrix} 1 & 0 & 1 \\ 0 & 1 & 1 \end{matrix} \begin{matrix} 1 \\ 1 \end{matrix} \right]$ | (S48) |
| --- | --- | --- |

(A = 1, B = 2, AB = 3, BA = 4), and the stoichiometric matrix of two selected independent reactions (Pekař and Samohýl, 2014; p.153) is

|  | $\left\Vert P^{p\alpha} \right\Vert=\left[ \begin{matrix} -1 & -1 & 1 \\ 0 & 0 & -1 \end{matrix} \begin{matrix} 0 \\ 1 \end{matrix} \right].$ | (S49) |
| --- | --- | --- |

Further

|  | $K_{1}=\frac{c_{AB,eq}}{c_{A,eq}c_{B,eq}}=\exp\left( \frac{\mu_{A}^{o}+\mu_{B}^{o}-\mu_{\mathrm{AB}}^{o}}{RT} \right); K_{2}=\frac{c_{BA,eq}}{c_{AB,eq}}=\exp\left( \frac{\mu_{\mathrm{AB}}^{o}-\mu_{\mathrm{BA}}^{o}}{RT} \right).$ | (S50) |
| --- | --- | --- |

In total, four affinities exist here, given by (see also Pekař and Samohýl, 2014; p. 181-182):

|  | $A^{1}=-\mu_{A}-\mu_{B}+\mu_{\mathrm{AB}}; A^{2} =-\mu_{\mathrm{AB}}+\mu_{\mathrm{BA}};$ | (S51) |
| --- | --- | --- |
|  | $B_{1}=\mu_{A}+\mu_{\mathrm{AB}}+\mu_{\mathrm{BA}}; B_{2} =\mu_{B}+\mu_{\mathrm{AB}}+\mu_{\mathrm{BA}}.$ | (S52) |

The basis vectors $\vec{g}$ (Pekař and Samohýl, 2014; p.153) and $\vec{f}$ (Pekař and Samohýl, 2014; p.152) are given here by $\vec{g}^{1}=-\vec{e}_{1}-\vec{e}_{2}+\vec{e}_{3};$ $\vec{g}^{2}=-\vec{e}_{3}+\vec{e}_{4};$ $\vec{f}_{1}=\vec{e}^{1}+\vec{e}^{3}+\vec{e}^{4};$ $\vec{f}_{2}=\vec{e}^{2}+\vec{e}^{3}+\vec{e}^{4}.$ The corresponding metric tensors (Pekař and Samohýl, 2014; p.154, 295-296) are:

|  | $\left\Vert g^{pq} \right\Vert=\left[ \begin{matrix} 3 & -1 \\ -1 & 2 \end{matrix} \right]; \left\Vert g_{pq} \right\Vert=\left[ \begin{matrix} 2/5 & 1/5 \\ 1/5 & 3/5 \end{matrix} \right];$ | (S53) |
| --- | --- | --- |

and

|  | $\left\Vert f^{\sigma\tau} \right\Vert=\left[ \begin{matrix} 3/5 & -2/5 \\ -2/5 & 3/5 \end{matrix} \right]; \left\Vert f_{\sigma\tau} \right\Vert=\left[ \begin{matrix} 3 & 2 \\ 2 & 3 \end{matrix} \right].$ | (S54) |
| --- | --- | --- |

The contravariant constitutive affinities can then be expressed from $B^{\sigma}=\sum_{\alpha} \sum_{\tau} \mu_{\alpha}S_{\tau\alpha}f^{\sigma\tau}$ as

|  | $B^{1}=\frac{3\mu_{A}-2\mu_{B}+\mu_{\mathrm{AB}}+\mu_{\mathrm{BA}}}{5}; B^{2}=\frac{-2\mu_{A}+3\mu_{B}+\mu_{\mathrm{AB}}+\mu_{\mathrm{BA}}}{5}.$ | (S55) |
| --- | --- | --- |

Note, that $B^{1}=(3B_{1}-2B_{2})/5$ and $B^{2}=(-2B_{1}+3B_{2})/5.$ Further

|  | $\mu_{A}=\frac{-2A^{1}-A^{2}+{5B}^{1}}{5}; \mu_{B}=\frac{{-2A}^{1}-A^{2}+{5B}^{2}}{5};$  $\mu_{\mathrm{AB}}=\frac{A^{1}-2A^{2}+{5B}^{1}+{5B}^{2}}{5}; \mu_{\mathrm{BA}}=\frac{A^{1}+3A^{2}+{5B}^{1}+{5B}^{2}}{5}.$ | (S56) |
| --- | --- | --- |

**4.2. First degree thermodynamic polynomial.** Retaining only the first degree term in (6), which was derived by a similar procedure to that shown above in 1.1, leads to the following polynomial:

|  | $\mathbf{J}=\mathbf{k}_{0001}\exp\left( \frac{-\mu_{\mathrm{BA}}^{o}}{RT} \right)\exp\left( \frac{A^{1}-2A^{2}+{5B}^{1}+{5B}^{2}}{5RT} \right)\left[ \exp\left( \frac{A^{2}}{RT} \right)-1 \right].$ | (S57) |
| --- | --- | --- |

Two conditions following from (2) are fulfilled trivially and identically in this case, viz. ${({\partial J_{1}}/{\partial A^{1}})}_{\mathrm{eq}}={({\partial J_{2}}/{\partial A^{1}})}_{\mathrm{eq}}=0.$ The third condition ${({\partial J_{2}}/{\partial A^{2}})}_{\mathrm{eq}}\leq0$ gives the second result in (28). The last condition is

|  | $\left[ \left( {\partial J_{1}}/{\partial A^{1}} \right)\left( {\partial J_{2}}/{\partial A^{2}} \right)-\left( {\partial J_{2}}/{\partial A^{1}}+{\partial J_{1}}/{\partial A^{2}} \right)^{2}/4 \right]_{\mathrm{eq}}\geq0.$ | (S58) |
| --- | --- | --- |

and from it the first result in (28) follows.

**4.3. Application of condition (2) to the second-degree thermodynamic polynomial.** This condition is applied to the simplified thermodynamic polynomial, (29), only. The derivatives ${\partial\mathbf{J}}/{\partial A^{2}}$ remain unchanged in equilibrium when compared to the first-degree case; this justifies the first condition in (30) also in this case. The other derivatives are as follows:

|  | $\left( \frac{\partial\mathbf{J}}{\partial A^{1}} \right)_{\mathrm{eq}}=-\mathbf{k}_{1100}\exp\left( \frac{-\mu_{A}^{o}-\mu_{B}^{o}}{RT} \right)\exp\left( \frac{A^{1}-2A^{2}+{5B}^{1}+{5B}^{2}}{5RT} \right)\frac{1}{RT}.$ | (S59) |
| --- | --- | --- |

One of the conditions resulting from (2), ${({\partial J_{1}}/{\partial A^{1}})}_{\mathrm{eq}}\leq0,$ thus gives with (S59) the second result in (30).

The last condition required to fulfil (2) calls for the modification of derivatives – they should be transformed into expressions with equilibrium concentrations. Using (S52) with chemical potentials, expressed as $\mu_{\alpha}=\mu_{\alpha}^{o}+RT \ln c_{\alpha},$ in equilibrium and (S50), we arrive at

|  | $\left( \frac{\partial\mathbf{J}}{\partial A^{1}} \right)_{\mathrm{eq}}=-\mathbf{k}_{1100}c_{A,eq}c_{B,eq}$ | (S60) |
| --- | --- | --- |

and

|  | $\left( \frac{\partial\mathbf{J}}{\partial A^{2}} \right)_{\mathrm{eq}}=\mathbf{k}_{0001}c_{BA,eq}.$ | (S61) |
| --- | --- | --- |

As required for a consistent treatment, result (S60) leads to the second result in (30), again, whereas result (S61) again gives the first condition in (30). Condition (S58) with (S60) and (S61) finally results in (31).

**4.4. Constraints on the traditional mass-action model.** The classical mass-action kinetic model runs as follows:

|  | ${dc_{A}}/{dt}={dc_{B}}/{dt}=-k_{1}c_{A}c_{B}+k_{2}c_{\mathrm{AB}}+k_{5}c_{\mathrm{BA}}-k_{6}c_{A}c_{B},$ | (S62a) |
| --- | --- | --- |
|  | ${dc_{\mathrm{AB}}}/{dt}=k_{1}c_{A}c_{B}-k_{2}c_{\mathrm{AB}}-k_{3}c_{\mathrm{AB}}+k_{4}c_{\mathrm{BA}},$ | (S62b) |
|  | ${dc_{\mathrm{BA}}}/{dt}=k_{3}c_{\mathrm{AB}}-k_{4}c_{\mathrm{BA}}-k_{5}c_{\mathrm{BA}}+k_{6}c_{A}c_{B}.$ | (S62c) |

The relationships between component and reaction rates in the presented methodology are generally $J^{A}=J^{B}=-J_{1},$ $J^{\mathrm{AB}}=J_{1}-J_{2},$ $J^{\mathrm{BA}}=J_{2}.$ In the simplified version given in the main text:

|  | $J^{A}=J^{B}=-k_{0001}^{1}c_{\mathrm{BA}}+k_{0001}^{1}K_{2}c_{\mathrm{AB}}-k_{1100}^{1}c_{A}c_{B}+k_{1100}^{1}K_{1}^{-1}c_{\mathrm{AB}},$ | (S63a) |
| --- | --- | --- |
|  | $J^{\mathrm{BA}}=k_{0001}^{2}c_{\mathrm{BA}}-k_{0001}^{2}K_{2}c_{\mathrm{AB}}+k_{1100}^{2}c_{A}c_{B}-k_{1100}^{2}K_{1}^{-1}c_{\mathrm{AB}}.$ | (S63b) |

Comparison of (S62) and (S63) leads to the following relationships:

|  | $-k_{1}-k_{6}=-k_{1100}^{1}; k_{2}=k_{0001}^{1}K_{2}+k_{1100}^{1}K_{1}^{-1}; k_{5}=-k_{0001}^{1};$  $k_{3}=-k_{0001}^{2}K_{2}-k_{1100}^{2}K_{1}^{-1}; -k_{4}-k_{5}=k_{0001}^{2}; k_{6}=k_{1100}^{2}$ | (S64) |
| --- | --- | --- |

and from them, for instance:

|  | $k_{2}=-k_{5}K_{2}+\left( k_{1}+k_{6} \right)K_{1}^{-1}; k_{3}=\left( k_{4}+k_{5} \right)K_{2}-k_{6}K_{1}^{-1}.$ | (32) |
| --- | --- | --- |

Thus, at most, four traditional rate constants are independent and determine (together with the equilibrium constants) the other two constants.

**References**

Pekař, M. (2009). Thermodynamic framework for design of reaction rate equations and schemes. Collect. Czech. Chem. Commun. 74*,* 1375–1401*.*

Pekař, M. (2010). Macroscopic derivation of the kinetic mass-action law. Reac. Kinet. Mech. Cat. 99, 29-35.

Pekař, M. (2016). Thermodynamic analysis of chemically reacting mixtures and their kinetics – example of a mixture of three isomers. ChemPhysChem 17, 3333-3341.

Pekař, M. and Samohýl, I. (2014). The Thermodynamics of Linear Fluids and Fluid Mixtures. Cham: Springer.

1. See also web page <http://www.fch.vut.cz/sapelite/index.html>. [↑](#footnote-ref-1)
